# Supplementary material for: Ethnic-Specific and UV-Independent Mutational Signatures of Basal Cell Carcinoma in Koreans
Source: Int J Mol Sci. 2025 Jul 19;26(14):6941. doi: 10.3390/ijms26146941 (PMC12295853; doi:10.3390/ijms26146941)
Supplement: Supplementary file 1 [file ijms-26-06941-s001.zip › ijms-3701585-supplementary.pdf]

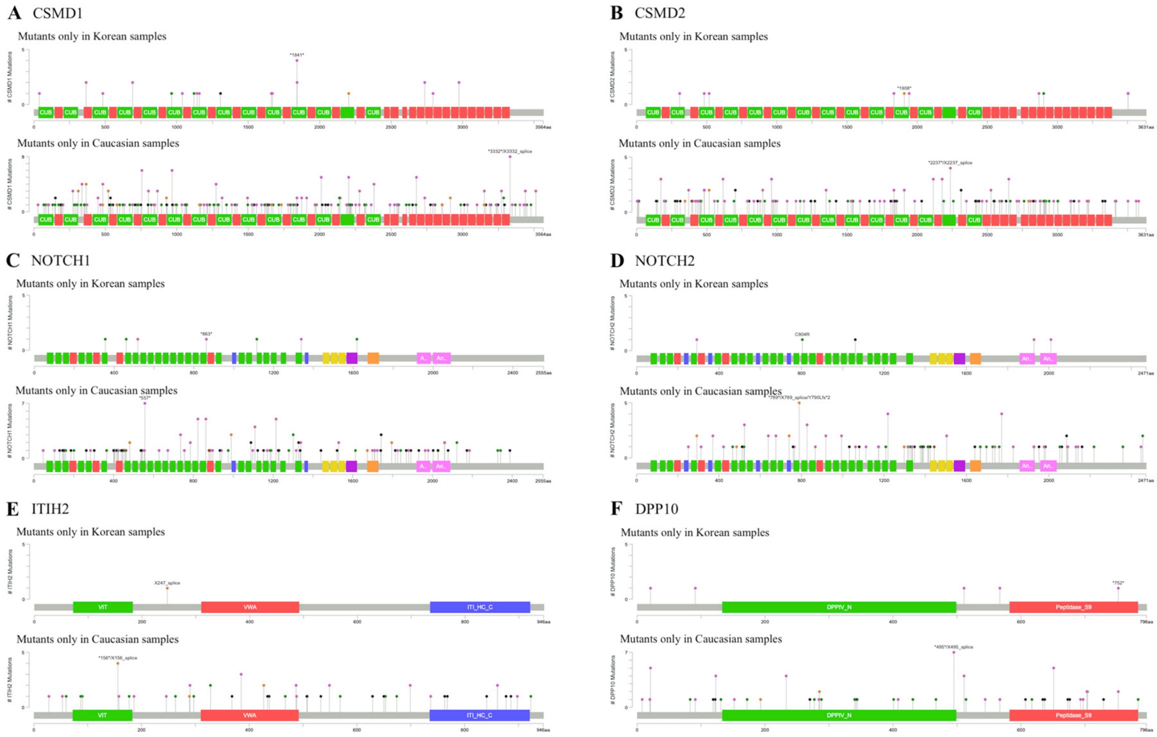

Figure S1. Different mutational profiles in Korean and Caucasian BCC groups. (**A**) Mutations of CSMD1. (**B**) Mutations of CSMD2. (**C**) Mutations of NOTCH1. (**D**) Mutations of NOTCH2. (**E**) Mutations of ITIH2. (**F**) Mutations of DPP10.

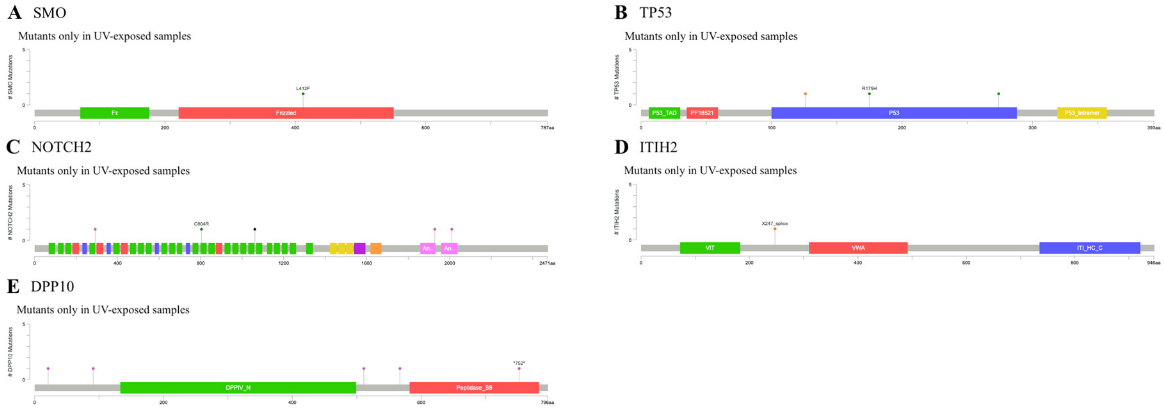

Figure S2. Mutation profiles in the BCCs of UV-exposed areas. (**A**) Mutations in SMO. (**B**) Mutations in TP53. (**C**) Mutations in NOTCH2. (**D**) Mutations in ITIH2. (**E**) Mutations in DPP10.

Table S1. Results of genome association analysis for the selected genes, TAS1R2 and ADCY10.
Table S2. Genetic variations in BCC marker genes identified exclusively in Koreans.
Table S3. Genetic variations in BCC marker genes identified in UV-exposed areas versus un-exposed areas.
